# Lower risk of hypoglycaemia and greater odds for weight loss with initiation of insulin detemir compared with insulin glargine in Turkish patients with type 2 diabetes mellitus: local results of a multinational observational study

**DOI:** 10.1186/1472-6823-14-61

**Published:** 2014-07-21

**Authors:** Taner Damci, Rifat Emral, Anne Louise Svendsen, Tanzer Balkir, Jiten Vora

**Affiliations:** 1 Department of Endocrinology, Diabetes and Metabolism, Cerrahpasa Medical School, Istanbul University, 34363 Istanbul, Turkey; 2 Department of Endocrinology and Metabolic Diseases, Faculty of Medicine, Ankara University, Ibn-i Sina Hospital, Ankara, Turkey; 3 Department of Biostatistics & Epidemiology, Novo Nordisk A/S, Søborg, Denmark; 4 Department of Clinical, Medical, & Regulatory Affairs, Novo Nordisk Saglik Urunleri Tic. Ltd. Sti., Etiler-Istanbul, Turkey; 5 Department of Diabetes and Endocrinology, Royal Liverpool University Hospital, Liverpool, UK

**Keywords:** Insulin detemir, Insulin glargine, Basal insulin, Type 2 diabetes, Weight loss, Hypoglycaemia

## Abstract

**Background:**

The purpose of this analysis is to evaluate the safety and effectiveness of insulin initiation with once-daily insulin detemir (IDet) or insulin glargine (IGlar) in real-life clinical practice in Turkish patients with type 2 diabetes mellitus (T2DM).

**Methods:**

This was a 24-week multinational observational study of insulin initiation in patients with T2DM.

**Results:**

The Turkish cohort (n = 2886) included 2395 patients treated with IDet and 491 with IGlar. The change in glycosylated haemoglobin (HbA_1c_) from the pre-insulin levels was -2.21% [95% confidence interval (CI) -2.32, -2.09] in the IDet group and -1.88% [95% CI -2.17, -1.59] in the IGlar group at the final visit. The incidence rate of minor hypoglycaemia increased in both groups from the pre-insulin to the final visit (+0.66 and +2.23 events per patient year in the IDet and IGlar groups, respectively). Weight change in the IDet group was -0.23 kg [95% CI -0.49, 0.02 kg], and +1.55 kg [95% CI 1.11, 2.00 kg] in the IGlar group. Regression analysis with adjustment for previously identified confounders (age, gender, duration of diabetes, body mass index, previous history of hypoglycaemia, microvascular disease, number and change in oral anti-diabetic drug therapy, HbA_1c_ at baseline and insulin dose) identified an independent effect of insulin type (IDet versus IGlar) with a risk of at least one episode of hypoglycaemia (odds ratio (OR): 0.33 [95% CI 0.21, 0.52], p <0.0001), and weight loss ≥1 kg (OR: 1.75 [95% CI 1.18, 2.59], p = 0.005), but not on HbA_1c_ (+0.05% [95% CI -0.15, 0.25%], p = 0.6).

**Conclusions:**

Initiation of basal insulin analogues, IDet and IGlar, were associated with clinically significant glycaemic improvements. A lower risk of minor hypoglycaemia and greater odds of weight loss ≥1 kg was observed with IDet compared with IGlar.

**Trial registration:**

NCT00825643 and NCT00740519

## Background

Many patients with type 2 diabetes mellitus (T2DM) ultimately require insulin to maintain glycaemic control. Nonetheless, there is still considerable debate regarding which insulin regimens are optimal to start the treatment cascade of the disease [1]. Basal insulin is a widely used treatment option for patients with T2DM at various stages of disease progression [2]. Basal insulin formulations have been continuously improved over the years and have become widely used as they have many pharmacodynamic and pharmacokinetic advantages over neutral protamine Hagedorn (NPH) insulin including a longer duration of action, reduced variable absorption profiles and a reduced marked peak effect [3]. Insulin detemir and insulin glargine are widely used as add-on therapies for patients not responding to oral anti-diabetic drug (OAD) regimens, but there are few studies directly comparing the two insulin analogues in routine care [4-6].

The primary objective of this international observational study, SOLVE™, was to evaluate the safety and effectiveness of once-daily insulin detemir or glargine in a real-life clinical setting in patients with T2DM failing OAD therapy. The following is a sub-analysis of the local SOLVE™ cohort in Turkey, the first observational study comparing the effects of once-daily insulin detemir with insulin glargine initiation as add-on therapy to OAD in patients with T2DM.

## Methods

### Study design

The present evaluation is a sub-analysis of the SOLVE™ study (clinical trial numbers NCT00825643 and NCT00740519). This study was a 24-week, non-interventional, international, multi-centre, open-label, prospective study of insulin detemir initiation in patients with T2DM treated with one or more OADs. SOLVE™ was conducted in 10 countries: Canada, China, Germany, Israel, Italy, Poland, Portugal, Spain, the UK and Turkey. The study was conducted in accordance with the Declaration of Helsinki and Guidelines for Good Pharmacoepidemiology Practice [7,8]. Ethical approval was obtained from local institutional review boards or independent ethics committees prior to commencement of the study in each of the participating countries [9]. Initiation of insulin therapy was entirely at the discretion of the treating physician according to local clinical practice, and the study enrolled and evaluated patients for whom this decision had already been made. Results from the global study cohort have been previously published [10,11].

In Turkey, the national regulatory requirements led to the inclusion of patients prescribed either insulin detemir or insulin glargine at least once daily, and thus provided an opportunity to compare the effects of the two insulin analogues in patients with T2DM. Data were collected during three routine clinic visits: a baseline visit immediately upon initiating treatment with once-daily insulin detemir or insulin glargine, an interim visit at 12 weeks, and a final visit at 24 weeks. Any procedures during the study period with regard to clinical care delivered were entirely at the discretion of the participating physician and the local practice in the investigating centre.

### Patients

The Turkish patient cohort was enrolled between April 2008 and October 2009. The inclusion and exclusion criteria for the global SOLVE™ study have been described elsewhere [11]. Patients already receiving one or more OADs and commencing treatment with either insulin detemir or insulin glargine within the previous 3 months could be enrolled in the study at the discretion of the investigator. Patients receiving insulin treatment for more than 3 months were excluded. Children below the age of 6 years and female patients who were pregnant, breast-feeding or intending to become pregnant within 6 months of the study initiation or who were not using adequate contraceptive methods were also excluded from the study. People with known or suspected allergy to insulin detemir or insulin glargine and those receiving glucose-lowering treatment other than diet, exercise or OAD before basal insulin therapy were also excluded. To limit selection bias, participating physicians were instructed to enrol patients on a consecutive basis until each site met the recruitment targets.

Patients could withdraw from the study at any time without giving any specific reason. Those patients who withdrew consent were not initiated on insulin at the baseline visit, and those who had an informed consent date after the baseline visit date, were excluded from the Turkish cohort.

### Endpoints

The primary goal for safety assessment was to evaluate the incidence of serious adverse drug reactions (SADRs), including major hypoglycaemic events, while using once-daily insulin detemir or once-daily insulin glargine in routine clinical practice. Safety evaluation also included the incidence of all daytime and nocturnal hypoglycaemic events and all other reported adverse drug reactions (ADRs).

ADRs were defined as any event for which a causal relationship to insulin detemir or insulin glargine was suspected, from the time the patient gave informed consent until the patient completed the study. The event was defined as serious if it resulted in any of the following: death, a life-threatening experience, in-patient hospitalization or prolongation of existing hospitalization for more than 24 hours, a persistent or significant disability/incapacity, a congenital anomaly/birth defect, or another important medical event that required medical or surgical intervention to prevent one of the outcomes listed in this definition. In this study, all episodes of major hypoglycaemia were considered to be SADRs.

All episodes of hypoglycaemia were self-reported. Major hypoglycaemia was defined as any hypoglycaemic event requiring assistance from a third party. Minor hypoglycaemia was defined as a blood glucose measurement <56 mg/dL (3.1 mmol/L) with or without symptoms. The period of recall for major hypoglycaemia and minor hypoglycaemia was 12 weeks and 4 weeks prior to the follow-up visit, respectively. Hypoglycaemic events were classified as nocturnal, if they occurred between bedtime and getting up the next morning.

Efficacy was assessed through the evaluation of glycosylated hemoglobin (HbA_1c_) and fasting blood glucose (FBG) (mean, change from baseline). Other secondary endpoints included: seven-point self-monitoring of blood glucose (SMBG) profile (pre- and post-breakfast, pre- and post-lunch, pre- and post-dinner, at night), body weight, waist circumference, waist-to-hip ratio, systolic and diastolic blood pressure, total cholesterol, low-density lipoprotein cholesterol, high-density lipoprotein cholesterol, triglycerides and insulin dose, and the use of OADs, antihypertensive drugs and lipid-lowering drugs.

### Statistical analysis

Patients using insulin detemir or insulin glargine at least once daily and reporting safety information to the physician were included in the analyses of adverse drug reactions (ADRs) and hypoglycaemia (Full Analysis Set [FAS]). Analyses of HbA_1c_, blood glucose and lipid profiles were based on a subset of patients with at least one FBG, HbA_1c_, weight measurement or record of hypoglycaemia at both baseline and final visit (Efficacy Analysis Set [EAS]).

Continuous variables are summarized with descriptive statistics (mean, standard deviation, 95% confidence interval (CI)). Categorical variables are reported in frequency tables (N,%). Statistical comparisons of pre- and post-insulin initiation values were performed with paired t-tests for continuous variables. Wilcoxon signed-rank test was used to compare the rates of hypoglycaemic events at baseline and final visit.

Regression models were used to evaluate the effect of insulin type on final-visit HbA_1c_ (general linear model), occurrence of at least one episode of hypoglycaemia from baseline to study end, and weight loss of at least 1 kg (logistic regression models). The weight model included all parameters identified as being significant predictors of weight loss ≥1 kg in the analyses of the global SOLVE™ cohort (data on file). This included gender, body mass index (BMI) categories (<25 kg/m^2^, 25 to <30 kg/m^2^, 30 to <35 kg/m^2^ and ≥35 kg/m^2^), number of OADs at baseline and baseline HbA_1c_. The HbA_1c_ and hypoglycaemia models included all parameters identified as being significant predictors either of HbA_1c_ at final visit or the occurrence of at least one episode of hypoglycaemia in analyses of the global cohort. These parameters included age categories (<50 years, 50–75 years in 5-year intervals, and ≥75 years), diabetes duration (in quartiles), BMI categories (as presented above), previous history of hypoglycaemia or microvascular disease, number and change in OAD therapy at the time of insulin initiation, HbA_1c_ at baseline and insulin dose (IU in quartiles) [12]. All regression models included the additional variable of the insulin type, to denote treatment with either insulin detemir or insulin glargine.

For the evaluation of HbA_1c_ and hypoglycaemia, two sensitivity analyses were performed. The first included a previous history of macrovascular disease in addition to the above-mentioned parameters. The second was a more basic model only adjusting for duration of diabetes, previous history of hypoglycaemia and baseline HbA_1c_. The results of the sensitivity analyses are available in Additional file 1.

All analyses used two-sided tests with the criteria set at α = 0.05.

## Results

A total of 2395 patients were enrolled in the insulin detemir group. Thirteen subjects were excluded for the following reasons: no insulin treatment (n = 4), informed consent date after baseline visit date (n = 8) and withdrew informed consent (n = 1). In the insulin glargine group, 491 patients were enrolled and three were excluded because of missing documentation of insulin treatment. The criteria for inclusion in the FAS were met by 78.3% (n = 1865) patients in the insulin detemir group and 70.9% (n = 346) in the insulin glargine group. The criteria for inclusion in the EAS were met by 68.4% (n = 1630) and 60.4% (n = 295) in the insulin detemir and insulin glargine groups, respectively.

A total of 571 (30.6%) people in the detemir group discontinued the study for the following reasons: lost to follow up (n = 355), OAD discontinued (n = 12), addition of short-acting insulin (n = 31), study drug used twice daily (n = 21), study drug discontinued (n = 27), other reasons (n = 49) and missing data (n = 106). In the glargine group, 155 (52.5%) people were withdrawn for similar reasons: lost to follow-up (n = 85), OAD discontinued (n = 4), addition of short-acting insulin (n = 9), study drug used twice daily (n = 1), study drug discontinued (n = 7), other miscellaneous reasons (n = 16) and missing data (n = 40). Patients may have had more than one reason for discontinuing the study.

The baseline characteristics of the patients of both groups are shown in Table 1. Baseline mean age, gender, weight, BMI, and previous medical history (including previous episodes of hypoglycaemia) were not significantly different between patients treated with insulin detemir and insulin glargine, and rates of hypoglycaemia were low in both groups (0.37 and 0.59 events per patient year, respectively). The group of patients treated with insulin detemir, however, had a significantly shorter duration of diabetes (8.1 vs. 8.6 years, p = 0.03), and a higher baseline HbA_1c_ (9.7 vs. 9.2%, [83 vs. 77 mmol/mol] p = 0.003) compared with patients treated with insulin glargine. The number and type of OADs used prior to insulin initiation also differed significantly between the groups, with a higher proportion of patients using a single OAD (30% vs. 26%) or >2 OADs (25% vs. 22%), in the group of patients initiating with insulin detemir (Table 2).

**Table 1:** Baseline characteristics of patients initiated on insulin detemir or insulin glargine: SOLVE™ Turkish cohort

	**Insulin detemir**	**Insulin glargine**	**p value**
N	2395	491	
Percentage completing the 24-week study (%)	76.2%	68.4%	
Age (years)	56.8 ± 10.2	56.6 ± 10.3	0.7979
Female (%)	57.2%	59.5%	0.3468
Duration of Diabetes (years)	8.1 ± 5.6	8.6 ± 5.4	0.0279
Weight (kg)	79.8 ± 13.9	78.5 ± 13.0	0.0606
BMI (kg/m^2^)	29.6 ± 4.8	29.6 ± 4.8	0.9541
Previous medical history (%)			
*Microvascular disease*	*30.0%*	*31.6%*	*0.4663*
*Macrovascular disease*	*21.3%*	*19.8%*	*0.4347*
*Hypoglycaemia*	*3.8%*	*3.9%*	*0.8940*
HbA_1c_ (%)	9.72 ± 1.74	9.24 ± 1.71	0.0033
HbA_1c_ (mmol/mol)	83 ± 19	77 ± 19	
FBG (mg/dl)	233 ± 75	221 ± 68	0.0989
FBG (mmol/l)	12.9 ± 4.2	12.3 ± 3.7	
Rate of minor Hypoglycaemia (events ppy)	0.37	0.59	0.0554
OAD Treatment at time of Insulin Initiation (%)			
Number of OADs			*0.0214*
*1 OAD*	*29.6%*	*26.3%*	
*2 OADs*	*45.0%*	*51.8%*	
*>2 OADs*	*25.4%*	*21.8%*	
Class of OAD			
*Metformin*	*81.9%*	*86.7%*	*0.0100*
*Sulphonylureas*	*55.5%*	*52.0%*	*0.1611*
*Glinides*	*21.6%*	*26.1%*	*0.0279*
*Thiazolidinediones*	*20.2%*	*18.8%*	*0.4745*
*α-glucosidase inhibitors*	*18.0%*	*10.8%*	*0.0001*
*DPP-IV inhibitors*	*1.8%*	*2.7%*	*0.2441*

Values are in mean ± SD or percentage.

**Table 2:** Key insulin treatment related endpoints of patients on insulin detemir and insulin glargine at final visit: SOLVE™ Turkish cohort (mean ± SD)

	**Insulin detemir**	**Insulin glargine**
HbA_1c_ (%)	7.48 ± 1.19	7.38 ± 1.24
HbA_1c_ (mmol/mol)	58 ± 13	57 ± 14
FBG (mg/dl)	145 ± 44	139 ± 38
FBG (mmol/l)	8.0 ± 2.4	7.7 ± 2.1
Weight (kg)	79.5 ± 12.6	79.7 ± 12.2
Rate of Minor Hypoglycaemia (events ppy)	1.08	2.56
Insulin Dose (U/kg)	0.30 ± 0.13	0.31 ± 0.14

### Serious adverse drug reactions (SADRs) and adverse drug reactions (ADRs)

SADRs, including major hypoglycaemia, were not reported during the observation period in either treatment group.

A single ADR was reported in the insulin detemir group (pruritus, skin and subcutaneous tissue disorder), and the relationship of this ADR to insulin detemir was determined as ‘probable’. It was documented that the condition recovered, and that neither the therapy, nor the insulin dose was modified.

### Hypoglycaemia

The incidence rate of minor hypoglycaemia increased significantly (p <0.001) in both groups from pre-insulin to final visit (+0.66 and +2.23 events per patient year in the detemir and glargine groups, respectively). Minor daytime hypoglycaemic events per patient year were 0.30 at baseline and 0.84 at final visit for insulin detemir, and 0.59 at baseline and 2.42 at final visit for insulin glargine. In the insulin detemir group, nocturnal hypoglycaemia occurred at a rate of 0.07 and 0.25 events per patient year at baseline and at final visit, respectively. In the insulin glargine group, the rate of nocturnal hypoglycaemia was 0 and 0.16 events per patient year at baseline and at final visit, respectively.

**Figure 1:**
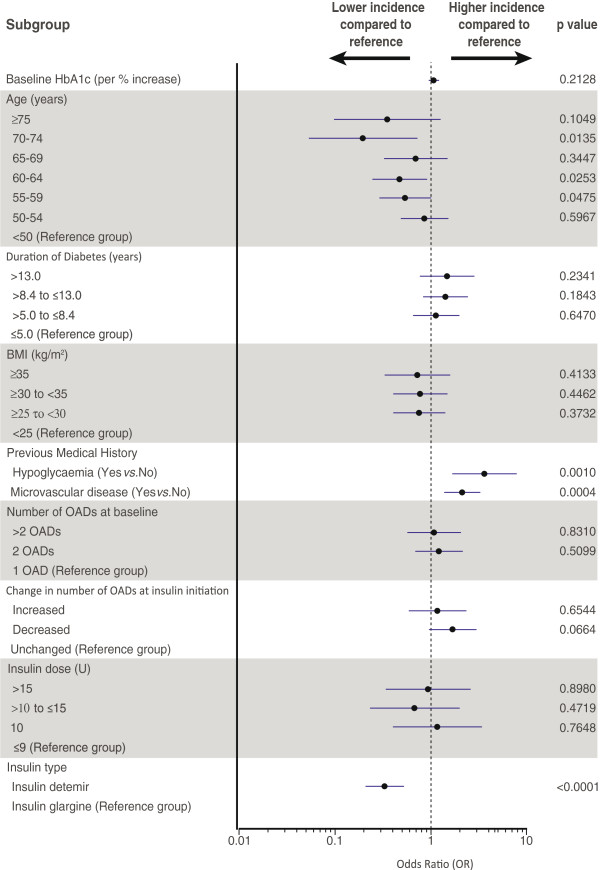
**Effect of insulin type (insulin detemir versus insulin glargine) on (a) hypoglycaemia during the study, (b) weight loss of ≥1 kg by final visit, and (c) final HbA**_**1c**_**.** Footnote: Error bars indicate 95% confidence intervals.

Insulin type was identified as an independent predictor of the occurrence of one or more episodes of hypoglycaemia during the study. After adjusting for the aforementioned confounders, insulin detemir had an odds ratio (OR) for hypoglycaemia of 0.33 [95% CI 0.21, 0.52, p < 0.001] relative to insulin glargine (Figure 1a). The relationship between insulin type and risk of hypoglycaemia during the study remained consistent in the sensitivity analyses (see Additional file 1).

### Weight, BMI and lipids

During the observational period, moderate weight loss was observed in patients treated with insulin detemir, with a mean change in weight of -0.23 kg [95% CI -0.49, 0.02]. In contrast, patients in the insulin glargine group gained weight, with a mean weight change of +1.55 kg [95% CI 1.11, 2.00]. Insulin type was identified as an independent predictor for weight loss ≥1 kg during the study after adjustment for known confounders (Figure 1b). Insulin detemir was associated with significantly greater odds of weight loss ≥1 kg (OR 1.75 [95% CI 1.18, 2.59; p = 0.005]) relative to insulin glargine.

Total cholesterol changed from 5.4 ± 1.1 mmol/l at baseline to 5.1 ± 1.0 mmol/l (change -0.4 mmol/l, [95% CI -0.5, -0.2], p < 0.001) in the insulin detemir group, and from 5.3 ± 1.1 mmol/l to 4.7 ± 0.9 mmol/l (change -0.6 mmol/l, [95% CI -0.9, -0.3], p < 0.001) in patients treated with insulin glargine. Triglycerides changed from 2.3 ± 1.3 mmol/l at baseline to 2.0 ± 1.0 mmol/l (change -0.3 mmol/l, [95% CI -0.4, -0.1], p < 0.001) in the insulin detemir group, and from 2.3 ± 1.2 mmol/l to 1.9 ± 0.9 mmol/l (change -0.4 mmol/l, [95% CI -0.6, -0.1], p = 0.01) in the insulin glargine group.

### Efficacy outcomes

Results showed no major difference in HbA_1c_ values during the observational period between insulin glargine and insulin detemir (Table 1). At the final visit, HbA_1c_ had changed significantly (p <0.001) from the pre-insulin levels by -2.21% [95% CI -2.32, -2.09] (-24 mmol/mol [95% CI -25, -23]) in the insulin detemir group, and by -1.88% [95% CI -2.17, -1.59] (-21 mmol/mol [95% CI -24, -17]) in the insulin glargine group. Fasting blood glucose (FBG) also changed significantly (p <0.001) from baseline to study end by -90 mg/dL [95% CI -97, -83] (-4.99 mmol/l [95% CI -5.38, -4.60]) in the insulin detemir group, and by -83 mg/dL [95% CI -98, -68] (-4.61 mmol/l [-5.42, -3.79]) in the insulin glargine group. All SMBG values improved significantly (p <0.05) over the study period in both patient groups (data not shown).

Regression analysis did not show insulin type to have an independent effect on the final HbA_1c_ value, with a difference of +0.05% [95% CI -0.15, +0.25%, p = 0.6] (+0.5 mmol/mol [95% CI -1.6, +0.25]) for insulin detemir relative to insulin glargine after adjustment for known confounders (Figure 1c). The relationship between insulin type and change in HbA_1c_ during the study remained consistent in the sensitivity analyses (see Additional file 1).

### Insulin dose

Baseline and final doses of insulin detemir and insulin glargine were similar. The mean baseline dose was 0.21 U/kg for both insulin types, and the mean dose at final visit was 0.30 U/kg for insulin detemir and 0.31 U/kg for insulin glargine.

## Discussion

This international multicentre observational study was performed to document the safety and effectiveness of once-daily insulin detemir and insulin glargine in patients with T2DM managed in a real-life clinical practice setting in Turkey. The total incidence of ADRs in the population of 2,886 Turkish patients was low; with only one ADR observed during the 24-week study, and no SADRs or major hypoglycaemic episodes, despite significant improvements in glycaemic control.

According to a prospective 6-year follow-up study in a representative sample of Turkish men and women, the annual incidence rate of T2DM was 11.0 and 12.4 per 1000 person-years in women and men, corresponding to 300,000 incident cases annually [13]. A recently published report estimated the prevalence of diabetes in Turkey in people aged over 20 years to be 16.5%, nearly half (45.5%) of whom were newly diagnosed [14]. Another multinational survey of 100 physicians in Turkey examining the perceived role of healthcare providers in tackling T2DM and the challenges they face, particularly regarding insulin treatment also found that most physicians had seen an increase in the number of T2DM patients over the previous 5 years, and almost all participating physicians agreed that the burden of diabetes was increasing [15]. Despite the high prevalence of diabetes in Turkey, most primary care physicians rarely initiate, modify or intensify insulin therapy, with the lack of experience and time to educate patients often being cited as the main barriers [15].

The Turkish part of the SOLVE™ study involved more than 200 participating physicians, and all treatment decisions were made at the discretion of the physician in accordance with patient’s clinical requirements. Thus, the treatment of patients within the study may be considered to reflect real-life clinical practice in Turkey. Baseline demographic data indicate inadequate glycaemic control and delayed insulin initiation. The mean baseline HbA_1c_ (approximately 9.5%) was well above the level of 7.0% recommended by the American Diabetes Association (ADA) [16]. Of all the countries participating in the SOLVE™ study, Turkey had the highest proportion of patients with a baseline HbA_1c_ ≥9.0% [10]. The mean diabetes duration of over 8 years at baseline also suggests delayed insulin initiation in this study population.

Basal insulin is a convenient and simple way to initiate insulin treatment in patients with T2DM. While there is general agreement that the currently available basal insulin analogue formulations are superior to human NPH insulin, in particular with respect to the risk of hypoglycaemia [17], there is no consensus as to which of the two available basal analogues should be recommended to initiate insulin treatment and what the potential differences are, if any, in patients with T2DM.

According to our knowledge, this is the first prospective non-interventional study to compare the effects of insulin detemir and insulin glargine in the real-life clinical environment. Several studies have compared either insulin detemir or insulin glargine with NPH insulin [18,19], but there are few studies directly comparing both basal insulin analogues [20,21]. Consistent with our findings, most head-to-head treat-to-target trials have not shown a significant effect of insulin detemir versus insulin glargine on HbA_1c_[20-22]. The only outlier is a recently published study by Meneghini et al. [23], which failed to confirm non-inferiority for insulin detemir versus insulin glargine. The lower than expected HbA_1c_ reductions in both groups (-0.48% for insulin detemir and -0.74% for insulin glargine) may be partly explained by the discontinuation of all OADs except metformin, without corresponding metformin dose adjustment and infrequent insulin dose titration.

Our safety results are consistent with other studies of once-daily insulin detemir initiation, both randomised controlled trials [24-26] and observational studies [27,28] in patients not responding to OAD therapy. These studies have consistently shown that insulin detemir has a good safety profile and a low incidence of hypoglycaemia. Randomised controlled trials comparing insulin detemir and insulin glargine have not reported significant differences in overall nocturnal and major hypoglycaemia rates [21,22], except for Meneghini et al. [23] where insulin detemir was associated with a significantly lower overall rate of hypoglycaemia. In this study, we report an independent effect of insulin type on the risk of minor hypoglycaemic episodes. As titration in this study was at the treating physicians’ discretion, the difference in the incidence of hypoglycaemia in favour of the insulin detemir group may be partly because of the varying up-titration between the two groups and the slightly higher HbA_1c_ values at final visit (7.48% vs. 7.38%) in patients treated with insulin detemir. However, reduced intra-patient glucose variability seen in patients treated with insulin detemir may also play a role [29-31]. Unlike some of the previous randomised controlled trials, where final insulin dose was found to be greater for insulin detemir compared with insulin glargine at the final visit [21-23], our data indicate similar end-of-study dose values between the two insulin analogues.

In the present study, insulin detemir also demonstrated a favourable weight-sparing effect, and was associated with higher odds (1.75-fold) of weight loss ≥1 kg compared with patients in the insulin glargine group. Actual mean weight change among insulin detemir patients was negative in contrast to insulin glargine-treated patients where the average weight increase was 1.5 kg. These results are consistent with previously reported randomised controlled trials and observational study results showing a trend toward less weight gain in patients administered with insulin detemir [20-22,27,28,32]. The mechanism for the weight-sparing effect of insulin detemir is still not fully understood, but might be due to differences in albumin binding, liver sensitivity, glucose variability and hypoglycaemia, or satiety signalling [33].

The SOLVE™ study has several important limitations that have been described elsewhere [11]. Because the study was not randomised, it is not possible to differentiate between the effects of treatment and other study or demographic variables on clinical outcomes, and therefore, the results of this study should be interpreted with caution. While the regression models were used to control for various known confounders, additional factors such as the speed of insulin up-titration, dose and type of combination OAD therapy and other variations in local clinical practice, may also have influenced the efficacy and safety of these two basal insulin analogues. Patients were recruited into the study after they were deemed to be candidates for once-daily insulin detemir or once-daily insulin glargine as add-on therapies to OADs based on the decision of the study physician according to local clinical practice. The percentage of patients lost to follow-up in the Turkish cohort (in both insulin detemir and glargine groups) was larger than for the total SOLVE™ cohort [11]. Both the recruitment and the loss to follow-up infer selection bias. In addition, whereas the recall of severe hypoglycemia appears to be preserved for a period of up to 1 year, the reliability of recall of episodes of mild hypoglycemia is unknown in patients with T2DM, and may be subject to recall bias [34]. The definitions of hypoglycemia (<56 mg/dL or 3.1 mmol/L) are consistent with other studies involving insulin detemir, and is a level at which autonomic symptoms of hypoglycemia are known to occur [35].

## Conclusion

The results from the SOLVE™ cohort in Turkey are consistent with previously reported randomised clinical trials and non-investigational study data of insulin detemir with regard to effective glycaemic control, low incidence of hypoglycaemia and a weight-sparing effect. Compared with T2DM patients initiated on insulin glargine in Turkey, insulin detemir was associated with a similar level of glycaemic control, but a lower risk of hypoglycaemia and greater odds of weight loss, after correction for a number of known confounders. This observational study provides useful additional information on the implementation and benefits of long-acting insulin analogues in a real-life clinical setting of Turkish patients with T2DM.

## Competing interests

Authors TD and JV have received financial support from Novo Nordisk to attend meetings to discuss the design, analysis and interpretation of the results of the SOLVE™ study.

TD has received consulting fees from Astra Zeneca, Bristol Meyers Squibb, Merck, Novo Nordisk and Sanofi-Aventis. RE has received consulting fees from Novo Nordisk. JV serves on advisory boards with Abbott Diabetes Care, Bristol Meyers Squibb, Johnson and Johnson, Lilly Industries, Merck, Novo Nordisk, Sanofi Aventis and Takeda Industries. JV has also received payment for lectures for Bristol Meyers Squibb, Daiichi-Sankyo, Lilly Industries and Novo Nordisk. JV’s institution has received research grants from Lilly Industries and Novo Nordisk. ALS and TB are employees of Novo Nordisk.

## Authors’ contributions

TD and JV have been involved with the study from conception, providing input to the study design, protocol, pre-defined analyses and interpretation of global results. TD has also monitored the conduct of the study in Turkey. The manuscript outline was prepared during a meeting with all authors; and all authors have given subsequent input to the drafts of this manuscript, and have reviewed and approved all content. ALS performed and/or reviewed all statistical analyses.

## Authors’ information

TD was the national principal investigator for this study.

## Pre-publication history

The pre-publication history for this paper can be accessed here:

http://www.biomedcentral.com/1472-6823/14/61/prepub

## Supplementary Material

Additional file 1 **The results of sensitivity analyses on the effect of insulin type (insulin detemir vs. insulin glargine) on (A) hypoglycaemia during the study, and (B) final HbA**_**1c**_**.** Click here for file
