# Supplementary material for: Lower risk of hypoglycaemia and greater odds for weight loss with initiation of insulin detemir compared with insulin glargine in Turkish patients with type 2 diabetes mellitus: local results of a multinational observational study
Source: BMC Endocr Disord. 2014 Jul 21;14:61. doi: 10.1186/1472-6823-14-61 (PMC4223563; doi:10.1186/1472-6823-14-61)
Supplement: Additional file 1 — The results of sensitivity analyses on the effect of insulin type (insulin detemir vs. insulin glargine) on (A) hypoglycaemia during the study, and (B) final HbA 1c . [file 1472-6823-14-61-S1.docx]

Supplementary Tables: The results of sensitivity analyses on the effect of insulin type (insulin detemir vs. insulin glargine) on (A) hypoglycaemia during the study, and (B) final HbA1c

A

| **Insulin type** | **Group comparison** | **Odds Ratio** | **95% Wald**  **Confidence Limits** | |
| --- | --- | --- | --- | --- |
| Insulin detemir vs Insulin glargine | Original Model^*^ | 0.33 | 0.21 | 0.52 |
|  | Restricted Model^†^ | 0.30 | 0.20 | 0.46 |
|  | Model with Macrovascular disease^‡^ | 0.34 | 0.22 | 0.53 |

B

| **Insulin type** | **Group comparison** | **HbA1c Effect (%)** | **95% Wald**  **Confidence Limits** | |
| --- | --- | --- | --- | --- |
| Insulin detemir vs Insulin glargine | Original Model^*^ | +0.05 | -0.15 | +0.25 |
|  | Restricted Model^†^ | +0.07 | -0.13 | +0.26 |
|  | Model with Macrovascular disease^‡^ | +0.04 | -0.16 | +0.24 |

^*^adjusted for the following parameters: age category (<50 years, 50-75 years in 5 year intervals, and ≥75 years), diabetes duration (in quartiles), body mass index (BMI) category (<25 kg/m^2^, 25 to <30 kg/m^2^, 30 to <35 kg/m^2^, and ≥35 kg/m^2^), previous history of hypoglycaemia or microvascular disease, number and change in OAD therapy at the time of insulin initiation, HbA1c at baseline and insulin dose (IU in quartiles); ^†^adjusted for duration of diabetes, previous history of hypoglycaemia, and baseline HbA1c; ^‡^as in original model with additional adjustment for a previous history of macrovascular disease.
